# Growth characteristics, optimal harvest timing, and quality assessment of three *Evodia* species cultivated in Japan

**DOI:** 10.1007/s11418-025-01886-7

**Published:** 2025-03-12

**Authors:** Koji Sugimura, Raju Aedla, Hiroyuki Fuchino, Osamu Iida, Takashi Watanabe

**Affiliations:** 1 Research Center for Medicinal Plant Resources, National Institutes of Biomedical Innovation, Health and Nutrition (NIBIOHN), 1-2 Hachimandai, Tsukuba, Ibaraki 305-0843 Japan; 2 Department of Medicinal Plant, Graduate School of Pharmaceutical Sciences, Kumamoto University, 5-1 Oe-Honmachi, Chuo-Ku, Kumamoto, 862-0973 Japan; 3 BVRIT Hyderabad College of Engineering for Women, Nizampet Road, Hyderabad, 500090 Telangana India; 4 Department of Pharmacy, Niigata University of Pharmacy and Medical and Life Sciences, 265-1 Higashijima, Akiha-Ku, Niigata City, Niigata 956-8603 Japan; 5 Global Center for Natural Resources Sciences, Kumamoto University, No. 5-1, Oe-Honmachi, Chuo-Ku, Kumamoto, 862-0973 Japan

**Keywords:** *Evodia* fruit, Immature fruit, Optimal harvesting period, Herbal medicine, Quality, Cultivation

## Abstract

Immature *Evodia* fruits are used in herbal medicine for their analgesic properties; however, determining the appropriate time for harvesting these fruits remains challenging. Here, we investigated the growth characteristics and optimal timing for collecting the immature fruits of three *Evodia* species—*E. rutaecarpa* (Juss.) Benth.*, E. officinalis* Dode*,* and *E. hupehensis* Dode—cultivated at the Tanegashima Division of the Research Center for Medicinal Plant Resources, Japan. Evodiamine and rutaecarpine content in the three species were measured across different collection seasons, and the relationship between time elapsed from the start of flowering and the levels of these active ingredients was determined. We found that the optimal time to collect the immature fruits of *E. officinalis* for use in herbal medicine was 2 weeks after flowering, when the fruit was heavier, contained more active ingredients, and had not yet dehisced. *E. rutaecarpa* fruits were heavier, contained more active ingredients, and retained their color (orange) until 3 weeks post-flowering. This suggests that the optimal collection time varied among species. Overall, *E. rutaecarpa* was the most suitable species for cultivation in Japan for use in herbal medicines because its optimal collection time was easier to determine, and its pericarp and seeds did not separate during drying. Therefore, to efficiently harvest *Evodia* fruits, cultivation methods should be optimized to leverage the specific growth characteristics of each species, with particular emphasis on accurately determining the optimal harvest time.

**Graphical abstract:**

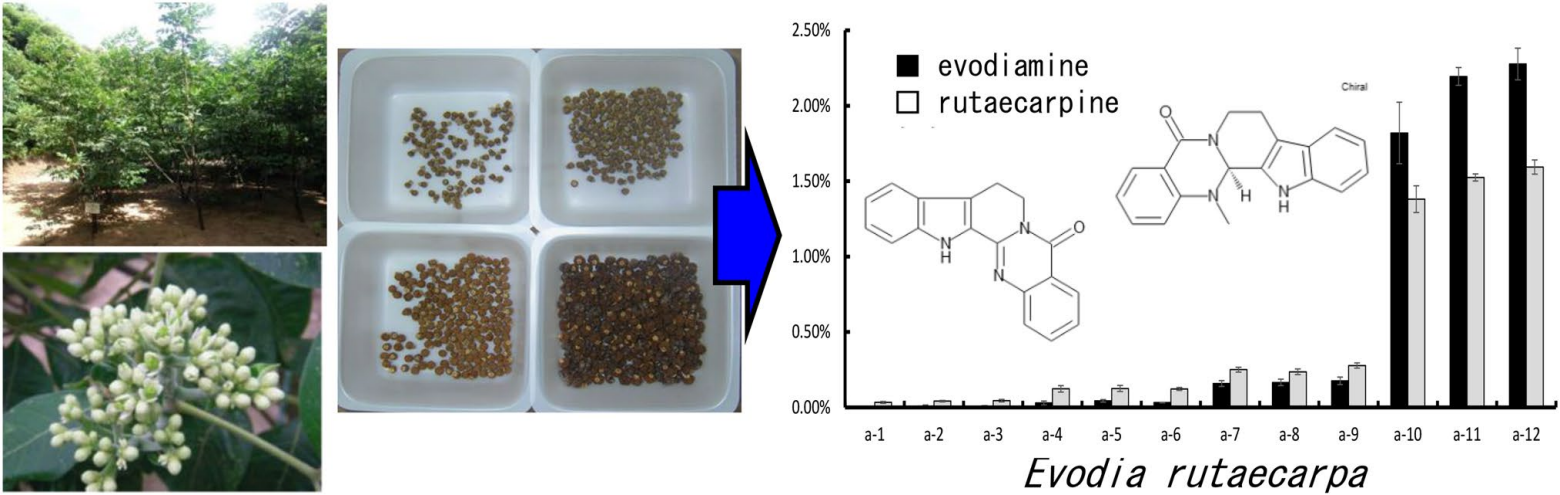

## Introduction

*Evodia* fruits are commonly used in herbal medicine to treat stomach ailments, diarrhea, vomiting, headache, and stomach pain, owing to their analgesic properties [1–3]. Immature *Evodia* fruits are a component of Kampo medicine formulations, such as Unkei-to and Goshuyu-to, which are important herbal medicines listed in the 18th Japanese Pharmacopoeia [4]. The original plant sources of these crude drugs include *Evodia rutaecarpa* Bentham (*Euodia ruticarpa* Hooker filius et Thomson) and *Evodia officinalis* Dode (*Euodia officinalis* Dode), which are deciduous shrubs belonging to the Rutaceae family and are widely distributed in China [5]. As only the female plants of *E. rutaecarpa* were introduced into Japan, the fruits do not produce seeds and gradually ripen, falling off after ripening without any apparent change in appearance [6, 7]. In contrast, both the male and female plants of *E. officinalis* are found in Japan. However, once seeds begin to form, they separate into the pericarp and seeds, making them unusable in herbal medicine. In particular, even if fruits that were not cracked at the time of harvest were collected, they often split during drying. Therefore, determining the appropriate time for collecting immature fruit from both species is extremely challenging.

Another plant, *Evodia hupehensis* Dode, is known to have a high yield because it has one of the largest inflorescences among the *Evodia* plants and produces a large number of flowers [5]. Although numerous studies have been conducted on the components of these fruits [8–12], no study has focused on the appropriate timing for harvesting the immature fruit from *Evodia* plants. To harvest crude drugs efficiently, understanding the optimal harvesting time for each *Evodia* species is necessary.

In this study, we conducted a comparative cultivation experiment of *E. rutaecarpa*, *E. officinalis*, and *E. hupehensis* on Tanegashima Island to differentiate their growth characteristics. We also aimed to determine the appropriate timing for harvesting immature fruits from plants of the three *Evodia* species. Furthermore, we assessed the quality of the three species of *Evodia* plants based on the component content at different collection times.

## Materials and methods

### Materials

Five-year-old *E. rutaecarpa*, *E. officinalis*, and *E. hupehensis* plants that were grown and cultivated in an open field at the Tanegashima Division of the Research Center for Medicinal Plant Resources (National Institute of Biomedical Innovation, Health and Nutrition, Osaka, Japan) from 2007 to 2011 were used in this study. The samples were stored at the Research Center for Medicinal Plant Resources, National Institutes of Biomedical Innovation, Health and Nutrition. Species identification was performed by Dr. Sugimura and confirmed by all co-authors. The voucher numbers were 0137-90TN for *E. rutaecarpa*, 0071F-01TN for *E. officinalis*, and 0088-06TN for *E. hupehensis*. The identification of *Evodia* species was determined by comparison with descriptions in Chinese Flora and by leaflet size, leaf axis color, leaf hair condition, and inflorescence shape [13]. *E. hupehensis,* known as a nectar source plant [14, 15], was examined as a candidate for high-yielding strains.

### Cultivation method

During cultivation, the spacing between the rows of different plant species was maintained at 2 m, with each line occupying a cultivation area of 40 m^2^, resulting in a total cultivation area of 120 m^2^. This configuration was consistently applied to ensure uniform growth conditions for all the plants under study. The plants were cultivated on the Tanegashima Division field (30°32′ N latitude, 130°27′ E longitude, and 88 m altitude). For fertilization, approximately 100 g each of compost, magnesium lime, and chemical fertilizer (8-8-8) were applied per hole as base fertilizer. Additional fertilizers, including nitrogen (10 kg), phosphoric acid (10 kg), and potassium (10 kg), were applied every spring.

### Fruit preparation method

The fruits were dried in shade for 3 days, followed by drying in a hot air dryer (EPFH-343-2 T; Isuzu, Yokohama, Japan) at 50 °C for 24 h.

### Investigation of growth characteristics

Average values of tree height, leaf length, number of branches, number of inflorescences, fruit diameter, 100 fruit weight, number of flowers per inflorescence, and yield per tree during the peak growth period of 5-year-old plants were measured (*n* = 5 for each species). A flowering survey was used to record the flowering start date and peak flowering period for all plants in 2011. Fully ripened fruits were separately collected 1, 2, and 3 weeks after flowering, and their dry weights were measured.

### Component analysis of immature fruit

The contents of evodiamine and rutaecarpine were measured using high-performance liquid chromatography (HPLC) in the immature fruits of *E. rutaecarpa*, *E. officinalis*, and *E. hupehensis*, collected at different times post-flowering.

### Analytical samples and reagents

Immature fruits were collected for analysis from the branches of three individuals of each of the three species (*E. rutaecarpa*, *E. officinalis*, and *E. hupehensis*) at four different stages: 1, 2, and 3 weeks post-flowering, and the ripe stage. The reagents used as standard substances included evodiamine (FUJIFILM Wako Pure Chemical Industries, Ltd., Osaka, Japan) and rutaecarpine (FUJIFILM Wako Pure Chemical Industries, Ltd.). The purities of evodiamine and rutaecarpine were > 95% and > 98%, respectively.

### Analysis methods

Dried fruits of *E. rutaecarpa* (0.5 g), *E. officinalis* (0.5 g), and *E. hupehensis* (0.2 g) were weighed and separately added to ~ 20 mL of methanol and then extracted using an ultrasonic device (AS ONE, Osaka, Japan) for 45 min. Thereafter, the samples were centrifuged at 2,000 rpm for 10 min, and the supernatant was transferred to a volumetric flask. Approximately 20 mL of methanol was again added to the residue, ultrasonicated, and centrifuged for another 45 min. The resulting supernatant was transferred to a volumetric flask, and methanol was added to bring the total volume to 50 mL. This extract was passed through a 0.45-μm filter and subsequently used as the sample for HPLC analysis. Simultaneously, 5.16 mg of evodiamine and 4.93 mg of rutaecarpine were separately weighed and added to methanol (25 mL) for use as the standard stock solution. A calibration curve was prepared using this stock solution and its serial dilutions (10- and 100-fold dilutions).

HPLC analysis was conducted using a Waters HPLC system with 717 plus Autosampler, 1525 Binary Pump, 2487 UV–Visible detector (Waters Corporation, Milford, MA, USA), and a TSKgel ODS–80 TM column (5 μm × 4.6 mm I.D. × 15 cm) (Tosoh, Tokyo, Japan). The HPLC conditions used were as follows: column temperature, 40 °C; detection wavelength, 254 nm; flow rate, 0.5 mL/min (for 0–25 min), ~ 1 mL/min (for 25–70 min); mobile phase, H_2_O/CH_3_CN/SDS/H_3_PO_4_ (500:500:5:0.1 v/v); and sample injection volume, 10 μL.

## Results

### Comparison of growth characteristics of the three Evodia species

**Fig. 1:**
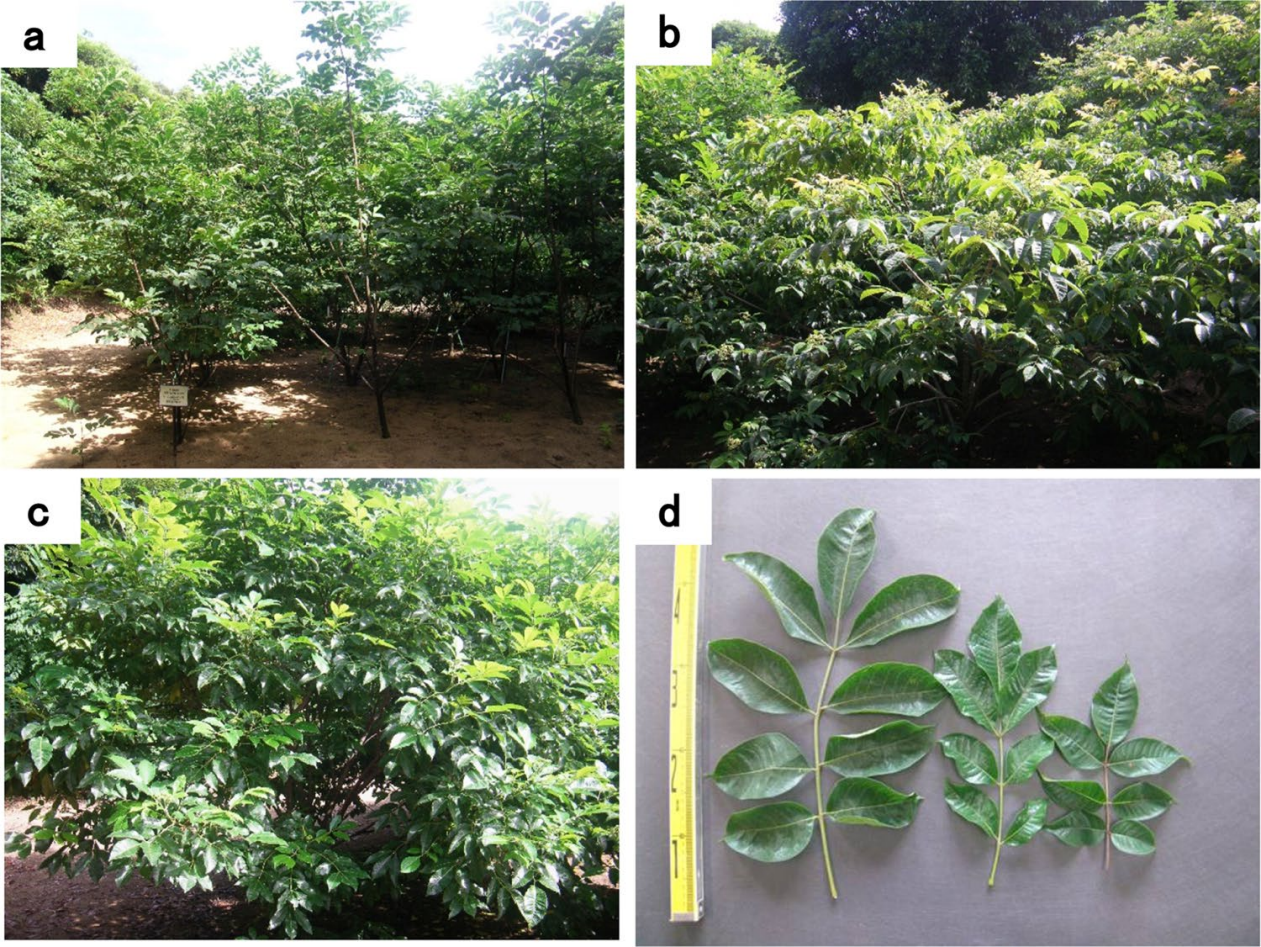
Whole plant and leaves of the three species of Goshuyu plants. (**a**) *Evodia rutaecarpa*, (**b**) *E. officinalis*, (**c**) *E. hupehensis*, and (**d**) (left to right) leaves of *E. rutaecarpa*, *E. officinalis*, and *E. hupehensis*

Table 1 summarizes the growth characteristics of plants of the three *Evodia* species, including the tree height (m), leaf length (cm), number of branches, inflorescences, and fruits per inflorescence; fruit diameter (mm), dry weight of 100 fruits (g), and dry weight of fruits per tree (g). Table 2 shows the diameter of fresh fruits collected from plants of the three *Evodia* species after 1, 2, and 3 weeks of flowering and during the fruit ripening period. Figure 1a–d shows images of the whole plants and leaves of the three *Evodia* species.

**Table 1:** Growth characteristics of plants of the three *Evodia* species

Characters	*E. rutaecarpa*	*E. officinalis*	*E. hupehensis*	*p*-value
Tree height (m)	3.5 ± 0.4^a^	2.4 ± 0.4^b^	2.8 ± 0.4^ab^	< 0.01
Leaf length (cm)	40.7 ± 3.1^a^	27.4 ± 4.1^b^	30.1 ± 3.9^ab^	< 0.01
Number of branches	33.0 ± 13.2^a^	128.2 ± 13.1^ab^	222.6 ± 69.9^b^	< 0.01
Number of inflorescence	27.0 ± 4.9^a^	60.5 ± 9.8^ab^	87.3 ± 5.4^b^	< 0.01
Fruit diameter (mm)	5.4 ± 0.4^ab^	11.7 ± 1.2^a^	1.9 ± 0.2^b^	< 0.01
Dry weight of 100 fruits (g)	4.0 ± 1.0^a^	3.5 ± 0.3^ab^	0.5 ± 0.1^b^	< 0.01
Number of fruits per inflorescence	162.7 ± 6.6^a^	202.7 ± 6.9^ab^	332.0 ± 9.5^b^	< 0.01
Dry weight of fruit yield per tree (g)	94.7 ± 16.7^ab^	237.3 ± 20.7^a^	59.3 ± 4.1^b^	< 0.01

Data are shown as mean ± SD (*n* = 5 plants per species)

The significance of differences between the three groups was tested using the Kruskal–Wallis test

**Table 2:** Fresh fruit diameter according to harvest time in the olnats of the three *Evodia* species

Collection time	*E. rutaecarpa*	*E. officinalis*	*E. hupehensis*	*p*-value
1 week after flowering (mm)	4.5 ± 0.3^a^	3.4 ± 0.3^ab^	1.8 ± 0.1^b^	< 0.01
2 weeks after flowering (mm)	4.9 ± 0.3^ab^	6.9 ± 0.3^a^	2.1 ± 0.2^b^	< 0.01
3 weeks after flowering (mm)	5.4 ± 0.4^ab^	11.7 ± 1.2^a^	1.9 ± 0.2^b^	< 0.01
Ripe period (mm)	6.3 ± 4.9^a^	12.2 ± 1.2^b^	–	< 0.01

Data are presented as mean ± SD (*n* = 5 samples per species)

The significance of differences between the three groups was tested using the Kruskal–Wallis test

The significance of the difference between the two groups was tested by* t*-test

The tree height and leaf length values increased in the order *E. officinalis* < *E. hupehensis* < *E. rutaecarpa*. A significant difference was observed between *E. rutaecarpa* and *E. officinalis* (*p* < 0.01). The number of branches, inflorescences, and fruit sets per inflorescence increased in the order *E. rutaecarpa* < *E. officinalis* < *E. hupehensis*, with a significant difference observed between *E. rutaecarpa* and *E. hupehensis* (*p* < 0.01). Notably, the fruit diameter and dry weight of fruit yield per tree increased in the following order: *E. hupehensis* < *E. rutaecarpa* < *E. officinalis*, with a significant difference between *E. hupehensis* and *E. officinalis* (*p* < 0.01). Furthermore, the values for 100 fruit dry weight increased in the order *E. hupehensis* < *E. officinalis* < *E. rutaecarpa*, with *E. hupehensis* and *E. rutaecarpa* significantly differing (*p* < 0.01). Fresh fruit diameter increased in the order of *E. hupehensis* < *E. officinalis* < *E. rutaecarpa* until 1 week post-flowering and then in the order of *E. hupehensis* < *E. rutaecarpa* < *E. officinalis* from 2 weeks post-flowering to the fully ripe stage. Significant differences were observed between *E. rutaecarpa* and *E. hupehensis* 1 week post-flowering, between *E. officinalis* and *E. hupehensis* 2 and 3 weeks post-flowering, and between *E. rutaecarpa* and *E. officinalis* during the fruit ripening period (*p* < 0.01).

### Comparison of flowering start date and peak flowering period of the three Evodia species

**Fig. 2:**
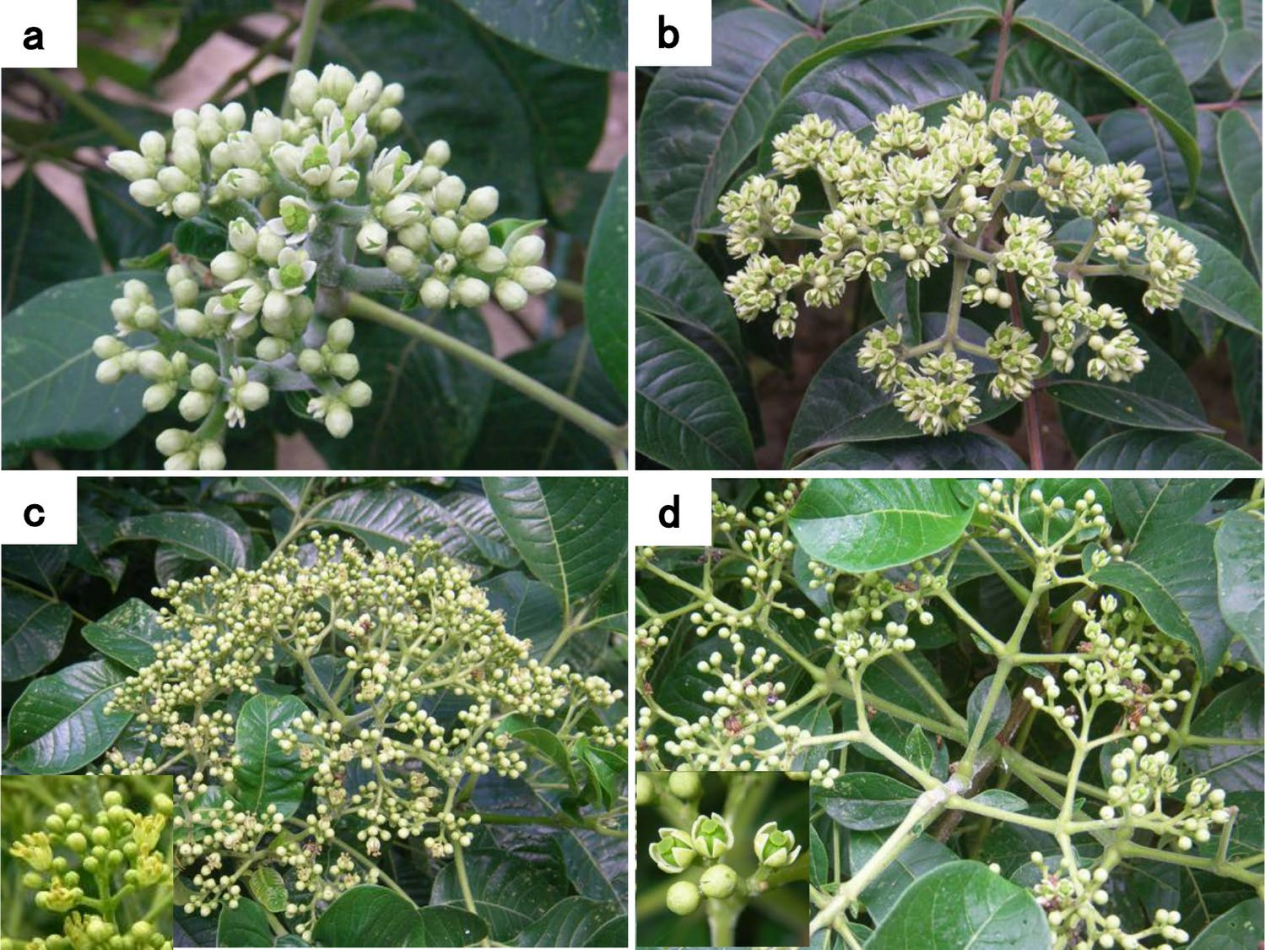
Flowers of the plants of the three *Evodia* species. (**a**) *E. rutaecarpa*, (**b**) *E. officinalis*, (**c**) *E. hupehensis* in the male stage, and (**d**) *E. hupehensis* in the female stage

Table 3 shows the flowering start dates and peak flowering periods of *E. rutaecarpa*, *E. officinalis*, and *E. hupehensis* plants. Figure 2a–d shows images of flowers of these species. Table 2 shows that both flowering parameters were in the following order (earlier to later): *E. officinalis* < *E. hupehensis* < *E. rutaecarpa*. Thus, the three *Evodia* species have different flowering periods, likely due to variations in growth characteristics, allowing them to avoid crossbreeding.

**Table 3:** Flowering start date and peak flowering period of the three *Evodia* species

Flowering category	*E. rutaecarpa*	*E. officinalis*	*E. hupehensis*
Flowering start date	Jul. 20, 2011	Jun. 24, 2011	Jul. 6, 2011
Peak flowering period	Jul. 25, 20 11	Jun. 30, 2011	Jul. 20, 2011

### Comparison of dry fruit weight per inflorescence among the three Evodia species

**Fig. 3:**
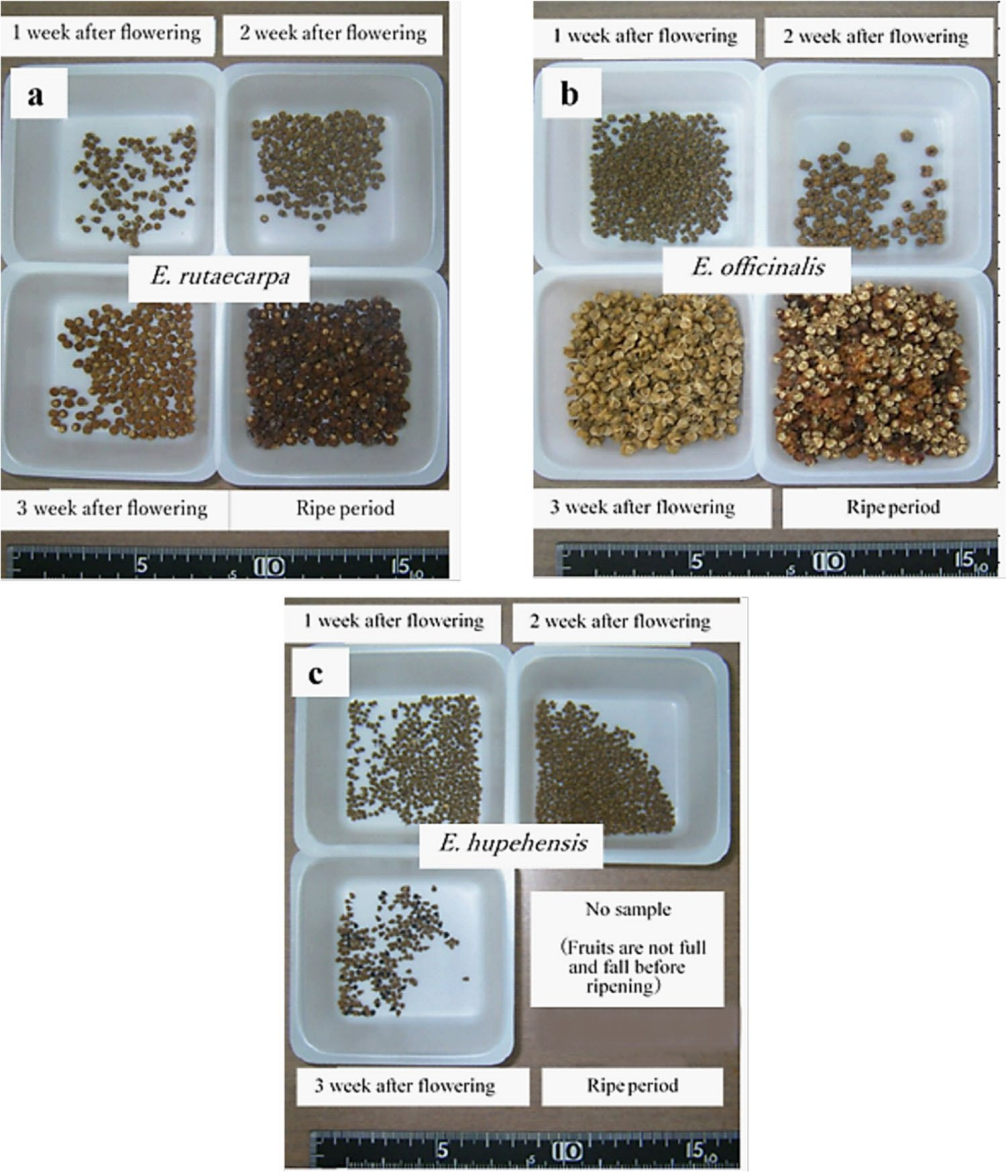
Dried fruits of the three *Evodia* species sorted by time of collection. (**a**) *E. rutaecarpa*, (**b**) *E. officinalis*, and (**c**) *E. hupehensis*

Table 4 shows the dry fruit weight per inflorescence and harvest date of the plants of the three *Evodia* species after 1, 2, and 3 weeks of the flowering and ripening periods. Figure 3a–c shows representative images of dry fruits of the three *Evodia* species, sorted by collection date. During all periods, the dry weight of fruit per inflorescence was in the order *E. hupehensis* < *E. officinalis* < *E. rutaecarpa*. After the ripe stage, the dry weight of *E. hupehensis* inflorescences was not measurable. The dried fruit weight of the three species did not significantly differ 1 week post-flowering. However, dried fruit weight significantly differed between *E. rutaecarpa* and *E. officinalis* 2 and 3 weeks post-flowering (*p* < 0.05). No significant differences in dried fruit weight were observed between *E. rutaecarpa* and *E. officinalis* at the fully ripe stage.

**Table 4:** Dry fruit weight per inflorescence and harvest date of the three *Evodia* species

Collection time	*E. rutaecarpa*	*E. officinalis*	*E. hupehensis*	*p*-value
1 week after flowering (g)	1.9 ± 0.7 (Aug. 1, 2011)	1.4 ± 0.5 (Jul. 7, 2011)	1.3 ± 0.5 (Aug. 2, 2011)	ns
2 weeks after flowering (g)	2.6 ± 1.2^a^ (Aug. 8, 2011)	2.2 ± 1.1^ab^ (Jul. 14, 2011)	1.0 ± 0.4^b^ (Aug. 9, 2011)	< 0.05
3 weeks after flowering (g)	4.0 ± 1.3^a^ (Aug. 15, 2011)	3.3 ± 1.5^ab^ (Jul. 21, 2011)	0.4 ± 0.4^b^ (Aug. 16, 2011)	< 0.05
Ripe period (g)	5.4 ± 0.6 (Nov. 4, 2011) (Approximately 16 weeks after flowering)	4.8 ± 0.6 (Sep. 3, 2011) (Approximately 9 weeks after flowering)	–	ns

Data are presented as mean ± SD (*n* = 3 samples per species)

The significance of differences between the three groups was tested using the Kruskal–Wallis test

The significance of the difference between the two groups was tested by* t*-test

Considering the changes in dry fruit weight by collection time, the dry fruit weight of *E. rutaecarpa* and *E. officinalis* tended to increase as the number of days after flowering increased. However, the fruit remained immature until 3 weeks post-flowering in *E. rutaecarpa* and up to 2 weeks post-flowering in *E. officinalis*. The dry weight of *E. hupehensis* fruits could not be measured at the ripe stage because they did not fully ripen or fall.

### Comparison of evodiamine and rutaecarpine content in immature fruits of the three Evodia species

**Fig. 4:**
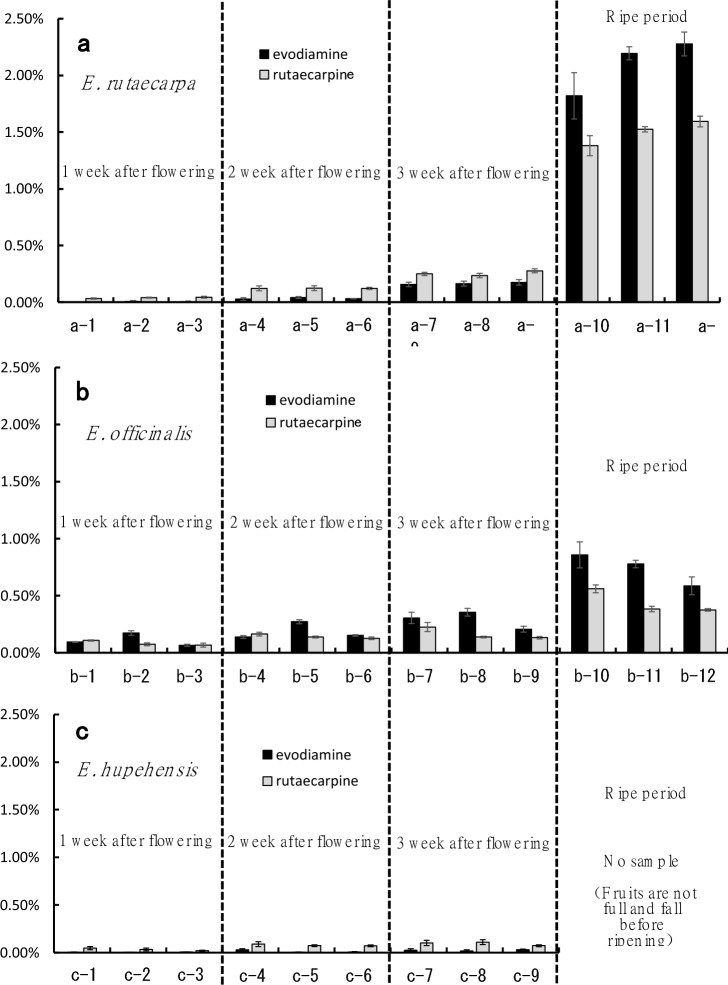
Contents of evodiamine and rutaecarpine according to harvest time in the three *Evodia* species. (**a**) *E. rutaecarpa*, (**b**) *E. officinalis*, and (**c**) *E. hupehensis* (*n* = 3 each), Values are the measurement error for the same sample, Vertical lines indicate SD

Figure 4a–c shows the evodiamine and rutaecarpine content of the immature fruits of the three *Evodia* species at different collection times (1, 2, and 3 weeks post-flowering and the ripe stage). One week post-flowering, fruit evodiamine content was 0–0.01% in *E. rutaecarpa*, 0.07–0.17% in *E. officinalis*, and 0% in *E. hupehensis*. Two weeks post-flowering, fruit evodiamine contents were 0.03–0.04%, 0.14–0.27%, and 0–0.03% in *E. rutaecarpa*, E*. officinalis*, and *E. hupehensis*, respectively. Three weeks post-flowering, the contents were 0.16–0.18%, 0.21–0.36%, and 0.02–0.03% in *E. rutaecarpa*, *E. officinalis*, and *E. hupehensis*, respectively. At the fully ripe stage, fruit evodiamine contents were 1.82–2.28% for *E. rutaecarpa*, 0.59–0.86% for *E. officinalis*, and not measurable in *E. hupehensis*. Compared with the evodiamine content of commercially available *E. rutaecarpa* (0.19–0.37%) [16], our observed content was slightly lower 1 week post-flowering, roughly the same 2 and 2 weeks post-flowering, and approximately sevenfold higher at full ripeness. The evodiamine content of *E. officinalis* in the present study was slightly lower 1 week post-flowering, roughly the same 2 and 3 weeks post-flowering, and approximately sixfold higher at full ripeness than that of commercially available *E. officinalis* (0.21–0.28%) [16].

Fruit rutaecarpine content 1 week post-flowering was 0.03–0.04% in *E. rutaecarpa*, 0.07–0.11% in *E. officinalis*, and 0.02–0.04% in *E. hupehensis*. Two weeks post-flowering, the rutaecarpine contents were 0.12%, 0.13–0.16%, and 0.07–0.09% in *E. rutaecarpa*, *E. officinalis*, and *E. hupehensis*, respectively. Three weeks after flowering, the concentrations were 0.24–0.28%, 0.13–0.23%, and 0.07–0.11% in *E. rutaecarpa*, *E. officinalis*, and *E. hupehensis*, respectively. After the ripe stage, the rutaecarpine content was 1.38–1.59% for *E. rutaecarpa*, 0.38–0.56% for *E. officinalis*, and not measurable in *E. hupehensis*. Compared with the rutaecarpine content of commercially available *E. rutaecarpa* (0.09–0.20%) [16], our investigation revealed levels that were slightly lower 1 week after flowering, roughly the same 2 and 3 weeks after flowering, and approximately fourfold higher at full ripeness. The rutaecarpine content in the present study was slightly lower 1 week post-flowering, roughly the same 2 and 3 weeks post-flowering, and approximately twofold higher at full ripeness than that of commercially available *E. officinalis* (0.07–0.29%) [16].

Thus, the content of evodiamine in fruits tended to gradually increase in the order *E. hupehensis* < *E. rutaecarpa* < *E. officinalis* from 1–3 weeks after flowering, while that of rutaecarpine content increased in the order *E. officinalis* < *E. rutaecarpa* from 3 weeks post-flowering to the ripening stage.

These results demonstrate that the contents of evodiamine and rutaecarpine in the fruits varied by species, with the differences becoming particularly evident at the ripe stage. Furthermore, considering the evodiamine and rutaecarpine content ratio in these fruits, evodiamine tended to be higher in *E. officinalis* and *E. rutaecarpa*, whereas rutaecarpine was higher in *E. hupehensis*. Additionally, we observed that the content of evodiamine and rutaecarpine increased with the number of days elapsed since flowering, indicating that the longer the time from flowering, the higher the content of both compounds. Particularly, the content was extremely higher at the fully ripe stage than that at other periods after flowering. For instance, *E. rutaecarpa* at the fully ripe stage had approximately 13.1-fold more evodiamine and approximately 5.9-fold more rutaecarpine than that at 3 weeks post-flowering. When fully ripe, the amount of evodiamine and rutaecarpine was approximately 2.5- and 2.7-fold higher than that at 3 weeks post-flowering.

Moreover, when examining the component composition by the harvest time of the fruit, the evodiamine content of *E. officinalis* tended to be almost equal to or slightly higher than that of rutaecarpine from 1 week after flowering until the fully ripe stage. Nevertheless, the component composition did not significantly change throughout the entire harvest period. Contrastingly, *E. rutaecarpa* showed a tendency for rutaecarpine to be slightly higher than evodiamine until the fruit was ripe, but thereafter, the evodiamine content was clearly higher than the rutaecarpine content, confirming a change in the component composition.

## Discussion

To efficiently produce and collect crude drugs, comprehending the unique characteristics of each plant species and determining their optimal harvest period is essential [17–19]. While determining the harvest period of immature *Citrus unshiu* fruits based on fruit color is straightforward, doing so for *Zanthoxylum piperitum* is more difficult as the fruit splits during drying [20–23]. Similarly, the optimal time for harvesting immature *Evodia* fruits is difficult to determine because the fruits split during drying [1, 2]. The results of the current study revealed that the optimal time to collect immature *E. officinalis* fruits was 2 weeks after flowering, when the fruit was heavy and contained a high percentage of active compounds, particularly evodiamine and rutaecarpine. In contrast, the optimal time to collect immature *E. rutaecarpa* fruits was 3 weeks after flowering, highlighting that the collection period differed depending on the species. Notably, *E. rutaecarpa* had the added advantage of the pericarp and seeds not separating because they were not fertile, as only female plants are cultivated in Japan. Furthermore, it also had the highest 100-fruit weight among the three species, as well as the highest evodiamine and rutaecarpine content. *E. officinalis* had high fruit-harvesting efficiency and a high dry fruit yield per tree; however, when the immature seeds began to ripen, its pericarp and seeds tended to separate during drying. Therefore, considering that split fruits did not meet the criteria for medicinal herbs, it became evident that immature *E. officinalis* fruits should be harvested as early as possible, within 2 weeks of flowering, despite the lower yield. The levels of active compounds in *E. officinalis* and *E. rutaecarpa* were highest in the mature fruits. However, as the herbal medicine *E. rutaecarpa* is traditionally produced from immature fruits [6, 24–26], determining the time when the fruits are not dehiscent is critical.

*E. hupehensis* not only had large numbers of inflorescences per tree but also produced a vast number of flowers per inflorescence. In addition, the flowers were confirmed to produce pollen and had stigmas, making *E. hupehensis* a high-yielding species; however, its fruits did not develop fully and fell before ripening, rendering it useless as a herbal medicine. Thus, *E. rutaecarpa* emerged as the most viable of the three *Evodia* species, owing to the ease of determining its optimal immature fruit-harvesting time and the added advantage of the pericarp and seeds not separating during drying. We also observed that the quality of *E. rutaecarpa* fruits was relatively high and consistent. Therefore, because of the introduction of only female *E. rutaecarpa* plants in Japan, which do not produce seeds and are easier to harvest, this species could be highly favored. *E. rutaecarpa* immature fruits not only have higher yields than those of *E. officinalis* and *E. hupehensis* but also consistently maintain higher and more stable active component contents. Thus, *E. rutaecarpa* was deemed the most suitable *Evodia* species for the cultivation and production of herbal medicines in Japan.

Moreover, if *Evodia* plants are used commercially to produce herbal medicines, the following must also be considered. Although *Evodia* plants are susceptible to scale insects, aphids, and spider mites, the damage is less than that of other common agricultural crops, and they have high disease resistance. *Evodia* plants can also be cultivated widely in warm regions and have a wide range of growth adaptability, making them relatively easy to cultivate. However, because our cultivation experiment was conducted at only one location, cultivation in another area with a different environment could alter the ingredient content depending on the soil and climate of that area. The details of such variations are currently unknown. For this reason, future comparative cultivation tests are necessary.

## Conclusion

To efficiently produce herbal medicines from *Evodia* fruits in Japan, it is crucial to comprehensively understand the unique characteristics of each species and employ cultivation methods that leverage these traits effectively. Particular emphasis should be placed on the accurate determination of the optimal harvest time. In this study, we deemed *E. rutaecarpa* as the most suitable *Evodia* species for the cultivation and production of herbal medicines in Japan.
